# Diagnostic Utility of the Immunohistochemical Expression of Serine and Arginine Rich Splicing Factor 1 (SRSF1) in the Differential Diagnosis of Adult Gliomas

**DOI:** 10.3390/cancers13092086

**Published:** 2021-04-26

**Authors:** Giuseppe Broggi, Lucia Salvatorelli, Davide Barbagallo, Francesco Certo, Roberto Altieri, Elena Tirrò, Michele Massimino, Paolo Vigneri, Elia Guadagno, Grazia Maugeri, Velia D’Agata, Giuseppe Musumeci, Marco Ragusa, Giuseppe Maria Vincenzo Barbagallo, Daniela Russo, Rosario Caltabiano

**Affiliations:** 1 Department of Medical and Surgical Sciences and Advanced Technologies “G.F. Ingrassia”, Anatomic Pathology, University of Catania, 95123 Catania, Italy; lucia.salvatorelli@unict.it (L.S.); rosario.caltabiano@unict.it (R.C.); 2 Department of Biomedical and Biotechnological Sciences, Section of Biology and Genetics Giovanni Sichel, University of Catania, 95123 Catania, Italy; dbarbaga@unict.it (D.B.); mragusa@unict.it (M.R.); 3 Department of Medical and Surgical Sciences and Advanced Technologies “G.F. Ingrassia”, Neurological Surgery, Policlinico “G. Rodolico-San Marco” University Hospital, University of Catania, 95123 Catania, Italy; cicciocerto@yahoo.it (F.C.); roberto.altieri.87@gmail.com (R.A.); gbarbagallo@unict.it (G.M.V.B.); 4 Department of Clinical and Experimental Medicine, Center of Experimental Oncology and Hematology, Policlinico “G. Rodolico-San Marco” University Hospital, University of Catania, 95123 Catania, Italy; ele_tir@yahoo.it (E.T.); michedot@yahoo.it (M.M.); vigneri.p@unict.it (P.V.); 5 Department of Advanced Biomedical Sciences, Pathology Section, University of Naples Federico II, 80131 Naples, Italy; elia.guadagno84@gmail.com (E.G.); daniela.russo@unina.it (D.R.); 6 Department of Biomedical and Biotechnological Sciences, Section of Anatomy, Histology and Movement Sciences, University of Catania, 95123 Catania, Italy; graziamaugeri@unict.it (G.M.); vdagata@unict.it (V.D.); g.musumeci@unict.it (G.M.)

**Keywords:** SRSF1, diagnosis, glioma, neuropathology, immunohistochemistry

## Abstract

**Simple Summary:**

Gliomas represent a wide group of central nervous system neoplasms, arising from the glial component of the central nervous system. They are generally sub-classified into astrocytomas, oligodendrogliomas, ependymomas and other rarer subtypes. Apart from morphological and molecular features, there are currently no specific markers for this heterogeneous group of tumors: thus, there is a need to identify more specific and useful markers to distinguish each histological subtype from the others. SRSF1 has been recently characterized as being functionally involved in gliomagenesis and it has been found that SRSF1 is increased in glioma tissues and its increased immunohistochemical expression among adult diffuse astrocytomas is positively correlated with histological grade. The aim of this study is to evaluate the immunohistochemical expression of the SRSF1 protein in a series of astrocytic and non-astrocytic adult gliomas, emphasizing its potential use in the differential diagnosis of these neuropathological entities.

**Abstract:**

Background: The aim of this study was to investigate the immunohistochemical expression and distribution of serine and arginine rich splicing factor 1 (SRSF1) in a series of 102 cases of both diffuse and circumscribed adult gliomas to establish the potential diagnostic role of this protein in the differential diagnosis of brain tumors. Methods: This retrospective immunohistochemical study included 42 glioblastoma cases, 21 oligodendrogliomas, 15 ependymomas, 15 pilocytic astrocytomas, 5 sub-ependymal giant cell astrocytoma and 4 pleomorphic xanthoastrocytomas. Results: Most glioblastoma (81%), oligodendroglioma (71%), sub-ependymal giant cell astrocytoma (80%) and pleomorphic xanthoastrocytoma (75%) cases showed strong SRSF1 immunoexpression, while no detectable staining was found in the majority of ependymomas (87% of cases) and pilocytic astrocytomas (67% of cases). Conclusions: The immunohistochemical expression of SRSF1 may be a promising diagnostic marker of astrocytomas and oligodendrogliomas and its increased expression might allow for excluding entities that often enter into differential diagnosis, such as ependymomas and pilocytic astrocytomas.

## 1. Introduction

The generic term “glioma” indicates a wide group of tumors of the central nervous system (CNS), arising from the glial component of CNS tissue which has a support and nutrition function for neurons [1,2]. Classically, based on the supposed specific cellular type of origin, gliomas are sub-classified into astrocytomas, oligodendrogliomas, ependymomas and other rarer subtypes, such as brain stem gliomas [3,4].

Although several rarer non-glial tumor entities have been described in the CNS [5], gliomas represent the most frequent form of primary intraxial CNS tumors, accounting for 24% of all adult brain and CNS neoplasms [1,6]; they encompass a broad spectrum of histological and biological entities with large differences in terms of histological appearance, prognosis and survival rates [1,6]. In the past, the classification of CNS tumors was solely based on their histological similarity to the presumed cell of origin [7]; it is easy to understand how the observation, with a light microscope, of hematoxylin and eosin (H&E)-stained sections of the tumor tissue, combined with immunohistochemical detection of specific proteins, represented the only validated diagnostic approach to these neoplasms. Assigning a histological grade was the only means of predicting the biological behavior of a CNS neoplasm. The 2016 World Health Organization (WHO) classification of CNS tumors introduced substantial changes to the previous morphology-based classification method [8,9,10,11]: the most important novelty was the introduction of “entity-defining” molecular alterations, such as chromosome 1p and 19q co-deletion for oligodendrogliomas [12]; the increasingly limited use of the diagnosis of “oligoastrocytoma”, restricted to cases in which molecular tests cannot be performed or are not conclusive [13]; the identification of specific mutations with both prognostic and diagnostic value, including the mutational status of the isocitrate dehydrogenase 1 and 2 genes (*IDH1/2*) for adult astrocytomas [14,15]. In particular, IDH mutations represent the most important independent prognostic factor associated with a favorable outcome among adult diffuse gliomas, as IDH-mutant adult astrocytomas have a relatively better prognosis in terms of disease-free survival and overall survival than their IDH wild-type counterparts.

Circular RNAs (circRNAs) are a recently discovered wide ranging group of RNAs with biological functions that have not yet been fully clarified [16,17]. However, the expression of several circRNAs has been found to be deregulated in several human diseases, from neoplastic to degenerative conditions, suggesting an active role of circRNAs in their pathogenesis [16,17]. The molecular characterization of circSMARCA5 has recently been published, a circular RNA has been found to be downregulated in WHO grade IV glioblastoma (GBM) biopsies with respect to normal brain parenchyma; it acts as a decoy for the oncogenic serine and arginine rich splicing factor 1 (SRSF1) [18,19]. It has also been demonstrated that modulation of circSMARCA5 expression influences the splicing pattern of vascular endothelial growth factor A (VEGFA) in the U87MG cell line, used as a GBM in vitro model [18,19], stimulating the formation of the pro-angiogenic isoform of VEGFA. SRSF1 has been recently characterized as being functionally involved in gliomagenesis: in particular, this protein which normally shuttles between the nucleus (where, once phosphorilated, accumulates in the speakles, contributing to the assembly of the spliceosome) and the cytoplasm (where it interacts with other proteins) is upregulated in GBM and contributes to the aberrant splicing of a plethora of target pre-mRNAs [18,19]. Zhou et al. [20] recently found that SRSF1 is increased both in glioma tissues and cell lines, and its increased immunohistochemical expression is positively correlated with histological grade (from WHO grade II diffuse astrocytomas to WHO grade IV GBM), the Ki-67 index, and a predicted poorer prognosis in GBM patients. To the best of our knowledge, no data on the immunohistochemical expression of SRSF1 in other gliomas, different from diffuse astrocytomas, are available in the scientific literature.

The primary aim of this research study was to evaluate the differential immunohistochemical expression of SRSF1 on a cohort of both GBMs and other diffuse and circumscribed gliomas, which must be frequently included in the histopathological differential diagnosis of adult gliomas, emphasizing the potential diagnostic utility of this protein in neuropathological practice. Furthermore, we validated our immunohistochemical findings by performing a Western blot (WB) analysis on GBM cell lines.

Nevertheless, we investigated both the role of SRSF1 as a potential poor prognostic factor in GBM patients and its oncogenic function through correlations with other predictors of glioma malignant behavior, including cell migration, blood vascular microvessel density (MVD) and autophagy.

## 2. Materials and Methods

The study was conducted according to the guidelines of the Declaration of Helsinki, and was approved by the Catania 1 Ethics Committee, Santa Sofia 78 street, Catania, Italy (protocol code: 166/2015/PO; 17/12/2015). Cases were retrospectively retrieved from the pathology files of the Section of Anatomic Pathology, Department of Medical, Surgical, and Advanced Technologies “G.F. Ingrassia”, University of Catania. Only confirmed cases, both histologically and molecularly of IDH-wild type GBM (*n* = 42), IDH-mutant and 1p/19q co-deleted oligodendroglioma (*n* = 21), ependymoma (*n* = 15), pilocytic astrocytoma (PA) (*n* = 15), sub-ependymal giant cell astrocytoma (SEGA) (*n* = 5) and pleomorphic xanthoastrocytoma (PXA) (*n* = 4), diagnosed during the period between 2015 and 2018, were included in the study. Clinical data were obtained from the original pathological reports. Overall survival (OS) times were available only for 28/42 GBM cases. H&E-stained sections and multiple slides stained with several immunohistochemical antibodies were available for each case. All the H&E sections were reviewed by three pathologists (G.B., L.S. and R.C.) to further confirm the previously established diagnosis. Each case was tested for immunohistochemical analyses as previously described [21,22], using the standard streptavidin–biotin labeling technique and the LSAB kit (Dako, Glostrup, Denmark) with appropriate positive and negative controls. Sections derived from paraffin-embedded specimens were deparaffinized in xylene for 15 min, rehydrated, and treated with 3% H_2_O_2_ for 10 min to block endogenous peroxidase activity, followed by extensive rinsing in double-distilled water and further rinsing for 15 min in 0.01 M phosphate-buffered saline (PBS), pH 7.4. Deparaffinized sections were incubated with mouse monoclonal anti-SRSF1 antibody (Santa Cruz Biotechnology; sc-33652), diluted 1:50 in PBS. Microwave pretreatment was crucial to enhance the staining in all the samples examined. Accordingly, all sections were pretreated with citrate buffer (pH 6.0) and exposed to radiation in a microwave oven. To reduce the commonly seen non-specific immunoreactivity due to endogenous biotin, sections were pretreated with 10 mg/mL of ovalbumin in PBS, followed by 0.2% biotin in PBS, each for 15 min at room temperature. Bound antibodies were revealed by incubation with 3,3-diaminobenzidine (Sigma-Aldrich, St. Louis, MO, USA) in 0.01% H_2_O_2_ for 5 min at room temperature. Sections were then counterstained with hematoxylin, dehydrated, and then mounted. Unaffected gallbladder sections were used as external positive controls. Negative controls consisted of the omission of the primary antibody.

The function “roctab” was applied to perform receiver operating characteristic (ROC) analyses with GBMs, oligodendrogliomas and control classification data (PAs and ependymomas). All statistical analyses were carried out using Stata v14 (StataCorp, College Station, TX, USA). Kaplan–Meier analysis was performed to calculate associations between SRSF1 expression and the OS times obtained from GBM patients. For all comparisons, the significance level was set to *p* < 0.05 for differences among groups.

### 2.1. Evaluation of Immunohistochemical Expression of SRSF1

Immunohistochemical slides were evaluated by three pathologists (G.B., L.S. and R.C.) with no information on patient clinical data. The presence of brown chromogen within the cell nuclei was interpreted as positive SRSF1 staining. No cytoplasmic staining was observed in any case. Unaffected gallbladder tissue was used as the external positive control to check the validity of the immunoreaction. Evaluation of the different staining patterns of SRSF1 was performed as previously described [23,24,25]. Intensity of staining (IS) was graded on a 0–3 scale (0 = absent staining, 1 = weak staining, 2 = moderate staining, 3 = strong staining). Five categories (0–4) of percentage of SRSF1 immunopositive cells (Extent Score, ES) were identified: <5%; 5–30%; 31–50%; 51–75%; >75%. IS was multiplied by ES to obtain the immunoreactivity score (IRS); low (L-IRS) and high (H-IRS) expression of SRSF1 were defined as IRS < 6 and IRS ≥ 6, respectively.

### 2.2. Glioblastoma Cell Lines

Experiments were performed on human GBM cell line U87MG(HTB-14), obtained from the American Type Culture Collection (ATCC, Rockville, MD, USA). Cells were grown in Dulbecco’s modified Eagle’s medium (DMEM) supplemented with 10% of heat-inactivated fetal bovine serum (FBS), 100 U/mL penicillin and 100 μg/mL streptomycin (Sigma-Aldrich, Steinheim, Germany). Cells were incubated at 37 °C in a humidified atmosphere with 5% CO_2_. pcDNA3-FLAG and FLAG-tagged SRSF1 vectors were a kind gift from Dr. Karoline Ebbesen. Experiments were performed by using cells between the 5th and 10th passage.

### 2.3. Western Blot Analysis

Western blot analysis was performed according to the procedures previously described by Maugeri et al. [26]. Briefly, proteins were extracted with buffer containing 20 mM Tris (pH 7.4), 2 mM EDTA, 0.5 mM EGTA; 50 mM mercaptoethanol, 0.32 mM sucrose and a protease inhibitor cocktail (Roche Diagnostics, Monza, Italy). Protein concentrations were determined by the Quant-iT Protein Assay Kit (Invitrogen, Carlsbad, USA). Approximately 20 µg of protein homogenate were diluted in 2X Laemmli buffer (Invitrogen), heated at 70 °C for 10 min, separated on a Biorad Criterion XT 4–15% Bis-tris gel (BIO-RAD, CA, USA) by electrophoresis and then transferred to a nitrocellulose membrane (BIO-RAD). Blots were blocked using the Odyssey Blocking Buffer (Li-Cor Biosciences, Nebraska, USA) and probed with appropriate antibodies: mouse anti-SRSF1 (1:100; sc-33652, Santa Cruz Biotechnology, Heidelberg, Germany) and rabbit anti-β-tubulin (1:500; sc-9104, Santa Cruz Biotechnology). The secondary antibody goat anti-rabbit IRDye 800CW (cat #926-32211; Li-Cor Biosciences) and goat anti-mouse IRDye 680CW (cat #926-68020D, Li-Cor Biosciences) were used at 1:15,000. Blots were scanned with an Odyssey Infrared Imaging System (Li-Cor Biosciences).

### 2.4. Cell Migration Assay

Cell migration was assayed through Oris™ Cell Migration Assay (Platypus Technologies, Madison, WI, USA), as previously described [27]. Briefly, 3.5 × 10^4^ transfected cells/well were seeded in a 96-well migration plate with the stoppers placed. After 24 h of growth in a 5% FBS medium, the stoppers were removed, but remained in place in the pre-migration reference wells (T0) until the time of assay readout. The plate was incubated in a humidified chamber for 24 h (T24) to allow for cell migration. Stoppers were then removed from the reference wells and images were captured using a Fluorvert inverted microscope (Leitz, Wetzlar, Germany) and imported to Image J software v. 1.51 (National Institutes of Health, Bethesda, MD, USA) for data analysis.

### 2.5. Assessment of Blood Vascular MVD

MVD was evaluated by two pathologists (G.B. and R.C.), as previously described [19,28]. Briefly, vascular hotspots were identified on tissue sections stained with anti-CD31 immunohistochemical antibody (JC70A; working dilution 1:40; DAKO, Glostrup, Denmark) by a light microscope at 4x and 10x magnifications. MVD represented the total amount of vessels per mm^2^ (conversion factor: 1 mm^2^ = 4 high power fields (HPFs)). Areas with ≥50 of viable tumor tissue were counted; extensive necrosis, hemorrhage and desmoplasia were considered as exclusion factors. Each single stained endothelial cell and every lumen for long branched vessels and glomeruloid tufts were counted. Finally, small clusters of ≥2 staining endothelial cells within the same vascular structure were counted as a single vessel.

### 2.6. Assessment of Autophagy Activation

In order to investigate the activation of the autophagic process and its correlation with SRSF1, we studied the expression of two immunohistochemical antibodies anti-autophagy related 7 (ATG7) and anti-autophagy related 4 (ATG4) on GBM (*n* = 42) and oligodendroglioma (*n* = 21) cases, correlating this with SRSF1 levels. Sections for authophagy assessment were treated as above-described and the immunohistochemical results were evaluated, as for SRSF1, according to the parameters of IS, ES and IRS, as described above. Both ATG7 and ATG4 exhibited cytoplasmic staining; no nuclear positivity was detected [28].

## 3. Results

Clinico-pathological and molecular features as well as immunohistochemical findings are summarized in Table 1.

### 3.1. Clinico-Pathological, Immunohistochemical and Molecular Features of the Glioma Cohort

Among the 42 GBM cases, 19 patients (45%) were males and 23 (55%) were females (mean age: 63 years). As regards the specific anatomic site, 23 GBMs (55%) were located at the temporal lobe, 10 (24%) at the parietal lobe and 9 (21%) at the frontal lobe, three of which presented contralateral spread. All 42 GBM cases were histologically and molecularly classified as WHO grade IV IDH-wild type; neither IDH1/2 mutations nor ATRX mutations were identified at the immunohistochemical and molecular levels; no evidence of 1p/19q co-deletion was found; p53 immunohistochemical overexpression was found in only seven cases (17%), but no TP53 mutation was molecularly identified. O^6^-methylguanine DNA methyltransferase (MGMT) promoter were not hypermethylated in all cases. The mean OS of the 28 GBM cases for which follow-up data were available was 17 months. Among the 21 oligodendroglioma patients, 12 (57%) were males and nine (43%) were females (mean age: 59 years). All oligodendrogliomas were located supratentorially, 8/21 (38%) at the temporal lobe, 7/21 (33%) at the parietal lobe and the remaining 6 (29%) at the frontal lobe. Histologically, 15/21 (71%) were diagnosed as WHO grade II oligodendroglioma and 6/21 (29%) as grade III anaplastic oligodendroglioma. All oligodendrogliomas included in the study presented IDH-1 (R132H) mutations, confirmed both immunohistochemically and molecularly. The 1p/19q co-deletion was found in 21/21 cases (100%). Of the individuals affected by ependymoma, 9/15 patients (60%) were males and 6/15 (40%) were females (mean age: 56 years). A total of 11/15 (74%) ependimomas were localized supratentorially, 2/15 (13%) in the posterior fossa and 2/15 (13%) in the spinal cord. Histologically, 14/15(93%) were diagnosed as WHO grade II classic ependymomas, 1/15 (7%) as WHO grade I myxopapillary ependymoma. All ependymomas were positively stained with GFAP and at least focally with EMA (dot-like/ring-like staining). OLIG-2 was negative in 13/15 cases (87%). IDH1/2, TP53, ATRX gene were wildtype. No 1p/19q co-deletion was found in any case. Among the PA patients, 11/15 (73%) were males and 4/15 (27%) were females, with a mean age at diagnosis of 23 years. 14/15 tumors (93%) were located at the cerebellum and 1/15 (7%) at the spinal cord. Immunohistochemical and molecular profiles of these tumors were relatively homogenous, showing diffuse positivity for GFAP and OLIG-2, focal expression of synapthophysin and absence of IDH 1/2, ATRX, TP53 mutations and 1p/19q co-deletion. All PAs included in the study showed a molecularly proven KIAA1549-BRAF fusion. Of the affected by SEGA, 3/5 patients (60%) were males and 2/5 (40%) were females (mean age: 21 years); all tumors (5/5) were located in the wall of the lateral ventricles and were associated to tuberous sclerosis syndrome. Immunohistochemically, all SEGAs were diffusely stained with GFAP, s-100 and SOX-2; focal positivity for synapthophysin was seen in 1/5 cases. Neither IDH 1/2, ATRX, TP53, BRAFV600E mutations, nor 1p/19q co-deletions were found. Finally, among the PXA patients, 3/4 (75%) were females and 1/4 (25%) male, with a mean age at the diagnosis of 26 years; 3/4 PXAs (75%) were located at the temporal lobe and 1/4 (25%) at the parietal lobe. Histologically, three cases (75%) were diagnosed as WHO grade II PXA and one case (25%) as WHO grade III anaplastic PXA; neoplastic cells exhibited an immunopositivity for GFAP in 4/4 cases and for synapthophysin in 1/4 cases; extravascular positivity for CD34 was observed in 3/4 cases. BRAFV600E mutations were molecularly found in 2/4 PXAs.

### 3.2. Differential Immunohistochemical Expression of SRSF1 in Adult Gliomas

**Figure 1:**
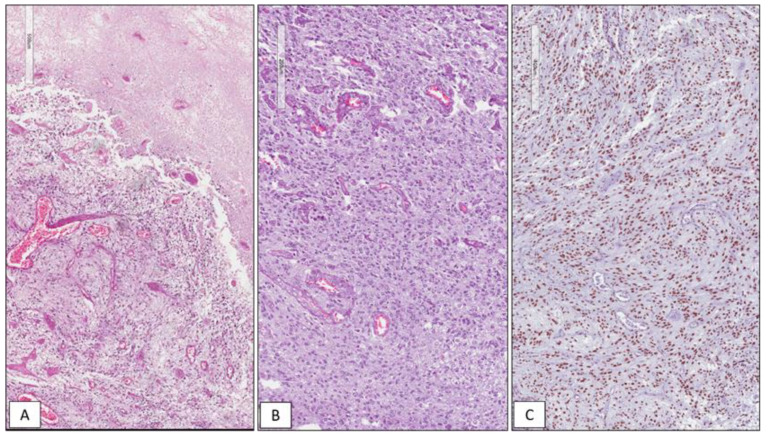
WHO grade IV IDH wild type glioblastoma. Histopathological examination showing a hypercellular glial tumor with extensive necrosis (**A**) and microvascular proliferation (**B**); high immunohistochemical nuclear expression of SRSF1 in a GBM tissue sample (**C**) (hematoxylin and eosin(**A**,**B**) and immunoperoxidase (**C**) staining; original magnifications 50×, 100× and 100×).

**Figure 2:**
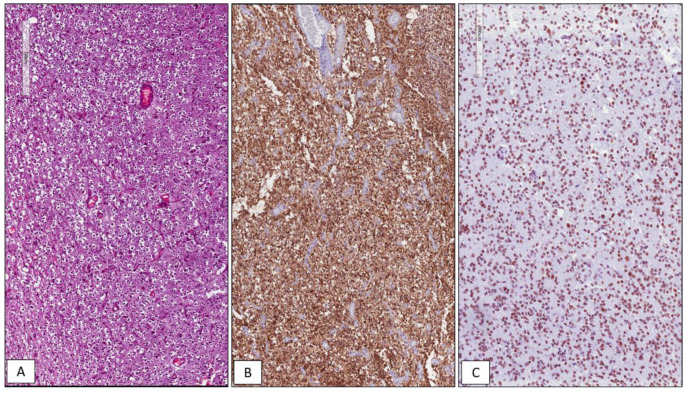
WHO grade II IDH mutant and 1p/19q co-deleted oligodendroglioma. Histopathological examination showing a moderately cellular glial tumor, composed of bland-looking round cells with a perinuclear clear halo with no evidence of necrosis and/or vascular proliferation (**A**); immunohistochemical positivity for IDH1 R132H reveals the presence of an IDH-1 gene mutation (**B**); high immunohistochemical nuclear expression of SRSF1 in WHO grade II oligodendroglioma (**C**) (hematoxylin and eosin (**A**) and immunoperoxidase (**B**,**C**) staining; original magnifications 100×, 100× and 100×).

**Figure 3:**
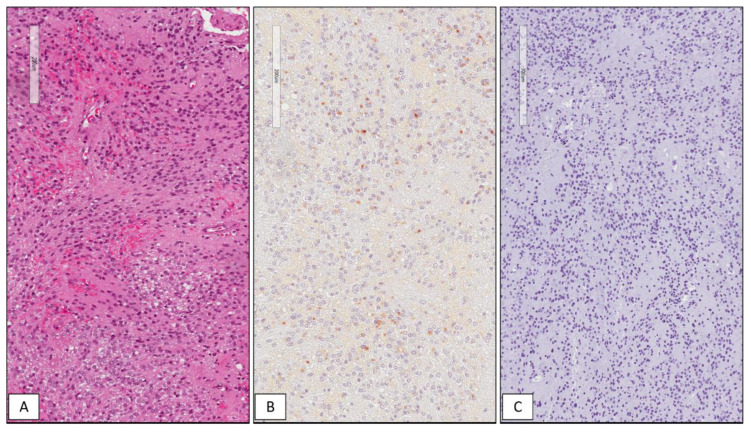
WHO grade II classic ependymoma. Histopathological examination showing a moderately cellular lesion, composed of small cells with rounded nuclei arranged in perivascular pseudorosettes with no evidence of anaplastic features (**A**); perinuclear “dot-like” staining for EMA highly suggestive for a diagnosis of ependymoma (**B**); absence of immunohistochemical expression of SRSF1 in WHO grade II classic ependymoma (**C**) (hematoxylin and eosin (**A**) and immunoperoxidase (**B**,**C**) staining; original magnifications 100×, 100× and 100×).

**Figure 4:**
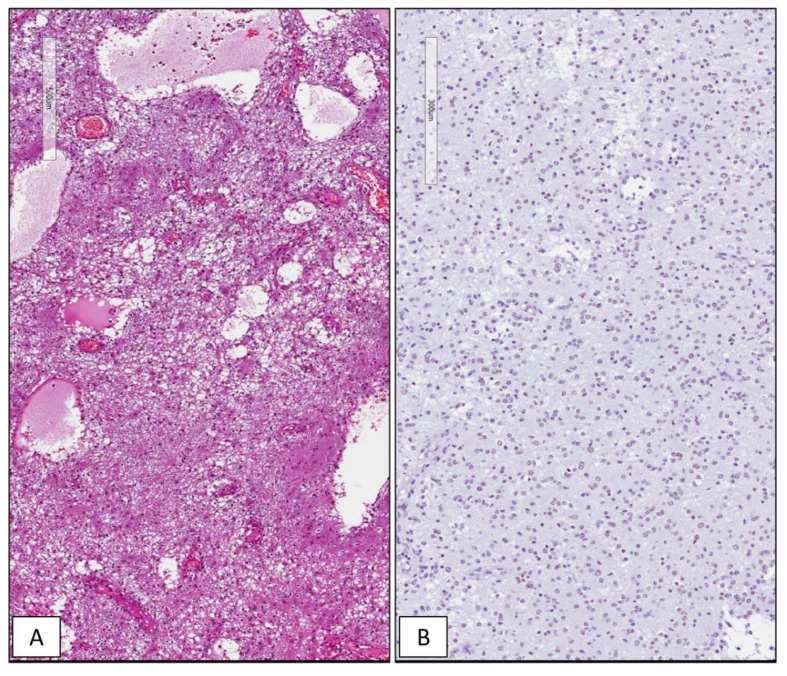
WHO grade I pilocytic astrocytoma. Histopathological examination showing the typical biphasic appearance consisting of compacted bipolar cells with Rosenthal fibers and multipolar cells embedded in a loose, myxoid and/or microcystic background (**A**); weak and focal nuclear immunohistochemical expression of SRSF1 in WHO grade I pilocytic astrocytoma (**B**) (hematoxylin and eosin (**A**) and immunoperoxidase (**B**) staining; original magnifications 100×).

**Figure 5:**
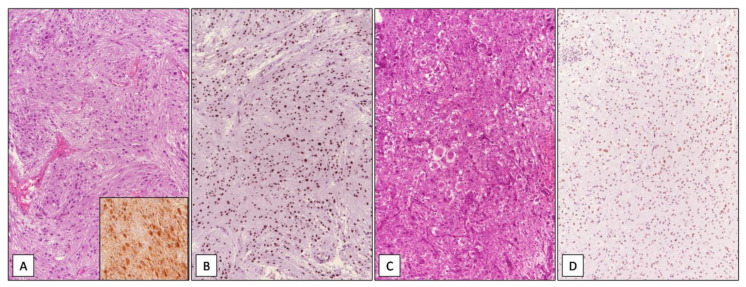
Sub-ependymal giant cell astrocytoma and pleomorphic xanthoastrocytoma. (**A**) Histopathological examination of sub-ependymal giant cell astrocytoma showing large polygonal to elongated cells with ganglioid/gemystocytic-like morphology and abundant eosinophilic cytoplasm; neoplastic cells typically exhibiting strong immunoreactivity for s-100 (insert); (**B**) strong and diffuse immunohistochemical expression of SRSF1 in sub-ependymal giant cell astrocytoma. (**C**) Histopathological examination of WHO grade II pleomorphic xanthoastrocytoma showing pleomorphic mono and multinucleated cells with focal cytoplasmic xanthomatous changes; (**D**) neoplastic cells were strongly and diffusely stained with SRSF1 (hematoxylin and eosin (**A**,**C**) and immunoperoxidase (**B**,**D**) staining; original magnifications 100×).

Among the 42 GBMs, 34/42 (81%) cases showed high immunohistochemical expression of SRSF1 (IRS ≥ 6) (Figure 1), whereas only in 8/42 (19%) low levels of immunohistochemical expression of SRSF1 (IRS < 6) were found. High SRSF1 levels (IRS ≥ 6) were found in 15/21 cases (71%) of oligodendrogliomas (Figure 2), whereas only 6/21 (29%) showed low expression (IRS < 6); all cases (6/6) of grade III anaplastic oligodendrogliomas had an IRS value ≥ 6. 2/15 (13%), ependymomas showed low SRSF1 levels (IRS < 6) and in the remaining 13/15 cases (87%), SRSF1 immunoexpression was completely absent (IRS = 0) (Figure 3). Similarly, low SRSF1 expression levels (IRS < 6) were found in 5/15 (33%) PAs (Figure 4) and in 10/15 cases (67%) no immunostaining (IRS = 0) was detected. Among the five SEGAs, 4/5 cases (80%) exhibited high SRSF1 immunoexpression (IRS ≥ 6) (Figure 5A,B), while a low level of SRSF1 immunoexpression was found in only one case (20%). Finally, 3/4 cases (75%) of PXA showed IRS values ≥ 6 (Figure 5C,D) and the remaining case (25%) had an IRS value < 6.

The specificity of SRSF1 for GBM was 100%, the sensitivity was 31.82% and the ROC was 0.66 (95% CI 0.56–0.76). The specificity of SRSF1 for oligodendroglioma was 100%, the sensitivity was 13.33% and the ROC was 0.57 (95%CI 0.48–0.65).

### 3.3. Correlation between Immunohistochemical Expression of SRSF1 and Prognosis

**Figure 6:**
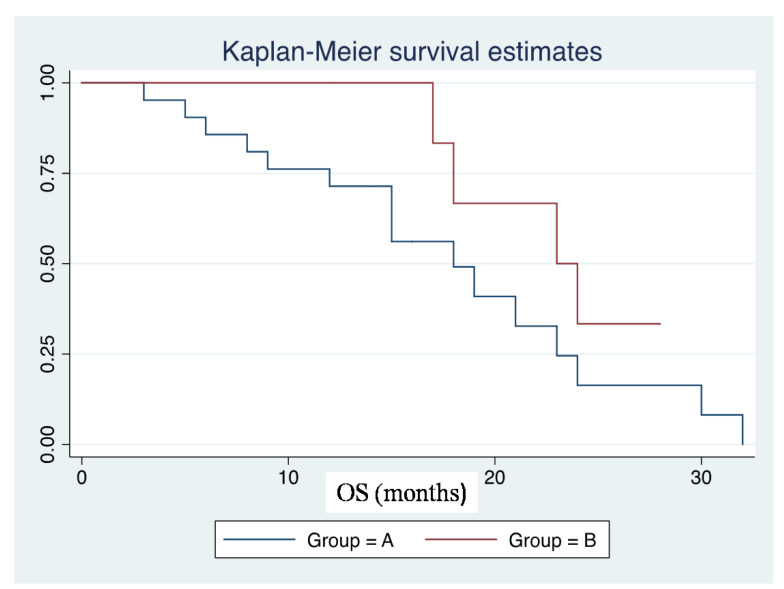
Group A (*n* = 21): patients affected by GBM with IRS_SRSF1_ values ≥ 6; Group B (*n* = 7): Patients affected by GBM with IRS_SRSF1_ values < 6.3.4.

Analysis of Kaplan–Meier survival curve showed that GBM patients with high immunoexpression of SRSF1 had lower OS times than the GBM group with low immunoexpression of this protein: median OS of patients with high immunoexpression of SRSF1 was 18.0 months (IQR 12.0–23.0), while patients with low SRSF1 levels exhibited median OS of 23.0 months (IQR 18.0–24.0) (*p* = 0.18). Figure 6 shows Kaplan–Meier survival estimates for the two groups.

### 3.4. Evaluation of SRSF1 Expression on GBM Cell Lines and Its Correlation with Cell Migration

**Figure 7:**
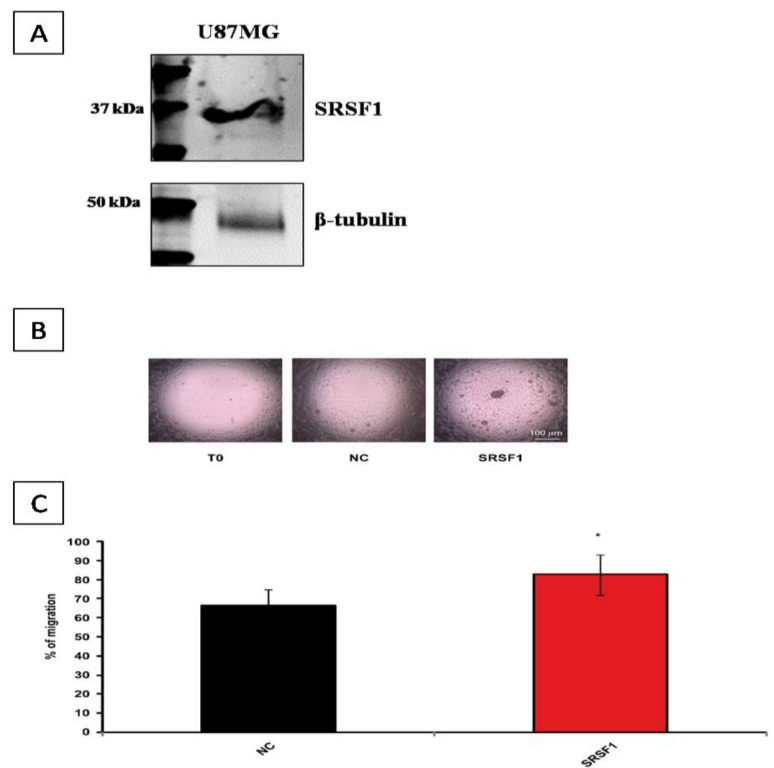
(**A**) Representative immunoblot of SRSF1protein on U87MG cells. β-tubulin was used as the loading control. (**B**) U87MG overexpressing SRSF1 migrated more than controls. Representative micrographs of migration of U87MG at T0 and at T24, in different experimental conditions: cells transfected with either empty pcDNA3 vector (NC) or SRSF1 expression vector. (**C**) Bar graph showing the mean ± standard deviation of the percentage (%) of NC or SRSF1-transfected U87MG cell migration. The percentage of cell migration has been calculated as previously reported [27]. * *p*-value < 0.05 (*n* = 6, Student’s *t*-test vs. control).

The expression of SRSF1 protein was evaluated using human GBM cell lines by Western blot analysis. U87MG cell line showed high levels of SRSF1 protein (Figure 7A), confirming the findings by Zhou et al. [20]. In addition, U87MG cells transfected with SRSF1 expression vector migrated more than controls (Figure 7B,C).

### 3.5. MVD Levels Positively Correlate with the Immunohistochemical Expression of SRSF1 on GBM Tissue Samples

**Figure 8:**
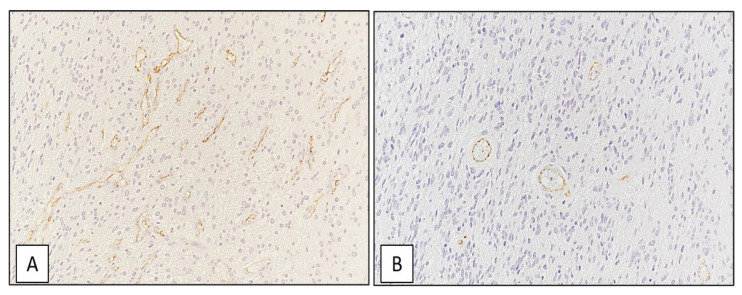
GBM tissue sample. High (**A**) and low (**B**) MVD count on GBM tissue (immunoperoxidase staining; original magnifications 200×).

The median value of MVD on the whole cohort of GBM cases (*n* = 42) was 93/mm^2^. As expected and as previously reported by our research group [19], a positive correlation between MVD and SRSF1 immunoexpression was found: in particular, GBM cases with high SRSF1 immunohistochemical expression (IRS ≥ 6) exhibited higher MVD values than those with SRSF1 IRS < 6 (median values: 114/mm^2^ vs. 67/mm^2^) (Figure 8A,B). These findings confirmed the previously reported data as regards the pro-angiogenic function of SRSF1.

### 3.6. Immunohistochemical Assessment of Autophagy and Its Correlation with SRSF1

**Figure 9:**
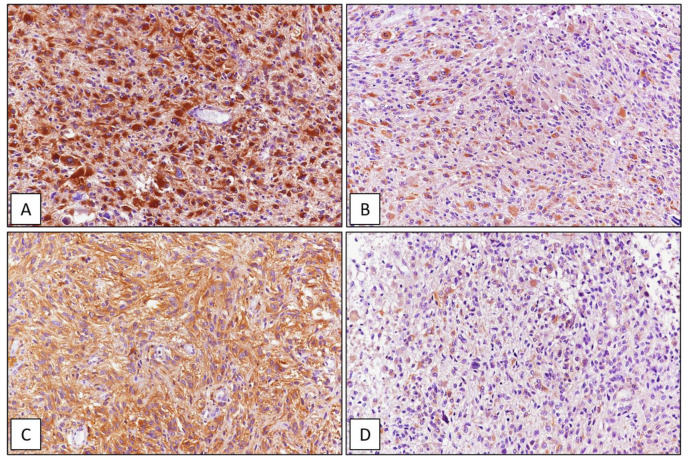
GBM tissue sample. High (**A**) and low (**B**) immunohistochemical expression of ATG7; high (**C**) and low (**D**) immunohistochemical expression of ATG4 (immunoperoxidase staining; original magnifications 200×).

**Figure 10:**
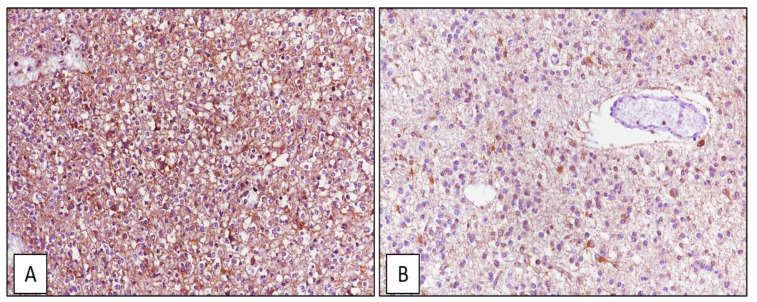
Oligodendroglioma tissue sample. High (**A**) and low (**B**) immunohistochemical expression of ATG4 (immunoperoxidase staining, original magnifications 200×).

Among the 42 GBM cases, high immunoexpression (IRS ≥ 6) of ATG7 was found in 32/42 cases (75%) (Figure 9A) and low immunoexpression (IRS < 6) in 10/42 (25%) (Figure 9B); 30/42 GBMs (71%) also exhibited high expression of ATG4 (Figure 9C), while low ATG4 levels were found in 12/42 cases (29%) (Figure 9D). Out of 21 oligodendrogliomas, ATG7 immunoexpression was high in 17/21 cases (81%) (Figure 10A) and low in 4/21 cases (19%) (Figure 10B); no staining for ATG4 was found in oligodendrogliomas. Interestingly, among the 34 GBM cases which exhibited high immunohistochemical expression of SRSF1, high ATG7 and ATG4 levels were found in 27/34 (79%) and 23/34 (68%) cases, respectively; the 15 oligodendrogliomas characterized by high SRSF1 immunoexpressions, also had high levels of ATG7 in 11/15 cases (73%).

## 4. Discussion

CircRNAs are a ranging wide subgroup of RNAs whose biology and active role in cellular metabolic “life” still remain largely unknown [16,17,18,19]. To date, in the scientific literature, there are numerous papers that describe how some specific circRNAs are effectively deregulated in several human degenerative and neoplastic diseases [16,17,18,19]. It is also known that splicing is the mechanism through which these RNAs originate [16,17,18,19]. It has been described that multiple circRNAs are highly expressed in normal and neoplastic tissue of the human brain; oncogenetic processes, including cellular differentiation and epithelial-to-mesenchymal transition, regulate their differential expression [16,17,18,19]. Barbagallo et al. [18,19] investigated the expression of 12 different types of circRNAs in both human unaffected brain parenchyma and CNS glial tumors and found that circSMARCA5 is the best example of downregulated circRNAs in WHO grade IV GBM specimens compared to normal cerebral tissue; particularly, circSMARCA5 seems to act as a tumor-suppressor RNA, negatively regulating cell migration through the RNA binding to protein SRSF1, which, in normal conditions, acts as an oncoprotein with a positive regulatory role on cell migration [18,19]. As a consequence, SRSF1 was found to be upregulated in GBM biopsies compared to the normal brain [18,19]. It has also been demonstrated that SRSF1 is involved in the splicing of vascular endothelial growth factor A (VEGF-A). The VEGF-A pre-mRNA splicing mechanism may alternatively generate both pro-angiogenic and anti-angiogenic isoforms [19]; it has been hypothesized that the downregulation of circSMARCA5, through the concomitant upregulation of SRSF1, leads to a switch in the proangiogenic–antiangiogenic ratio of VEGFA, resulting in angiogenic stimulation on GBM tissue [18,19]. The fact that patients affected by GBM with circSMARCA5 downregulation had a poorer prognosis than those with higher circSMARCA5 expression further supports these findings [18,19]. To the best of our knowledge, there are no papers in the scientific literature that have investigated the potential diagnostic role of SRSF1, assessed by immunohistochemistry, in the differential diagnosis of adult gliomas. We tested the immunohistochemical expression of SRSF1 in a cohort of patients affected by GBM and other glial neoplasms, including 21 oligodendrogliomas, 15 ependymomas, 15 Pas, 5 SEGAs and 4 PXAs. We found high SRSF1 expression levels in most oligodendrogliomas (15/21; 71%), SEGAs (4/5; 80%) and PXAs (3/4; 75%), an absence of SRSF1 staining in 13/15 (87%) ependymomas and in 10/15 (67%) PAs; the remaining ependymoma (2/15) and PA (2/15) cases showed weak SRSF1 expression.

The diagnosis of CNS neoplasms is one of the most challenging for pathologists: this is due both to the fact that neurosurgery is increasingly a surgical niche [29], many surgical pathologists are not familiar with CNS tumor diagnosis, and that there are very few morphological features and immunohistochemical markers with high specificity in neuropathology [30]. Many morphological and immunohistochemical features are commonly shared by several neoplasms: the most emblematic example is that—while in the past, the presence of round cells with a “fried-egg” appearance was considered a specific diagnostic clue of oligodendroglioma [31,32,33,34]—it is now well known that other entities, such as diffuse astrocytic tumors, PAs and ependymomas, may focally or extensively show the same oligodendroglioma-like morphology [35,36,37,38], and, conversely, many oligodendrogliomas may contain an astrocytic cellular component [31,32,33,34]. In this regard, the absence of specific pathological and immunohistochemical markers has led to the introduction of molecular biology as an essential tool for the diagnosis of CNS tumors [30,39]. Our purpose was to provide a new potential immunohistochemical marker to include in the antibody panel when approaching an adult glioma in pathology practice. As Zhou et al. already found that SRSF1 is immunohistochemically expressed with an increasing gradient in WHO grade II to IV astrocytic neoplasms, we preferred to include in our series gliomas in which the expression of SRSF1 had not yet been tested, such as oligodendrogliomas, ependymomas, PAs, SEGAs and PXAs.

The results obtained suggest a relevant diagnostic utility of the SRSF1 protein in distinguishing adult diffuse astrocytomas from ependymomas and PAs, the diagnosis of which may be particularly challenging when tumors arise in adults and in anatomic sites different from the cerebellum; we decided to include PA in the cohort of gliomas studied because, although it is not properly a diffuse adult glioma but a pediatric one, it is an entity that can arise and be diagnosed at any age, and therefore, must be frequently included in the differential diagnosis of low-grade adult gliomas.

As regards the diagnosis of oligodendroglioma, a potential application of SRSF1 would be in the distinction between oligodendrogliomas with astrocytic-like morphology and grade II and III adult astrocytomas, which may present a oligodendroglioma-like cellular component, as SRSF1 appears to be more diffusely expressed in the former and less expressed in the latter; interestingly, SRSF1 seems to be more constantly expressed in grade III anaplastic oligodendrogliomas than their grade II counterparts, as IRS values ≥ 6 were found in all grade III cases (6/6) included in our series. Further studies, involving a larger series of grade III anaplastic oligodendrogliomas, would be useful to better investigate the immunoexpression of SRSF1 among these neoplasms.

Based on our results, SRSF1 does not appear to be a reliable marker in distinguishing SEGA and PXA from their morphological mimickers; however, given both the glioneuronal phenotype and the presence of BRAFV600E mutations of PXA and the characteristic clinico-pathological features of SEGA, the diagnosis of these entities is usually more straightforward for the neuropathologists.

In addition, we found that SRSF1 may be also used as prognostic factor in GBM patients: Kaplan–Meier survival analysis showed that GBM patients who exhibited high immunoexpression of SRSF1 had lower OS times than those with low immunoexpression (median OS of 18.0 months vs. 23.0 months). SRSF1 expression was also correlated with other parameters of increased malignancy of gliomas, such as cell migration, MVD and autophagy activation. We found that U87MG GBM cells transfected with SRSF1 expression vector migrated more than controls and that GBM tissue samples with high SRSF1 immunohistochemical expression had higher MVD value than those with SRSF1 low expression (median values: 114/mm^2^ vs. 67/mm^2^), confirming the pro-angiogenic function of SRSF1.

Autophagic cascade is a catabolic process through which the cells remove their damaged organelles and proteins [28]. The role of autophagy in cancer still remains controversial, as, in the early stages of tumorigenesis, it seems to play a tumor suppressor role, while in advanced stages, a tumor suppressive function [28]. The immunohistochemistry represents, at present, the best way to study the autophagy on tissue samples [40]. More than 30 autophagy related proteins are targetable by immunohistochemical antibodies. In particular, we tested ATG7 and ATG4 on the whole cohort of GBM and oligodendroglioma cases. ATG7 is located on chromosome 3 and encodes an E1-like activating enzyme that plays a key role for intracellular transport [28]. ATG4 encodes for a key Cys-protease crucial for the autophagosome-mediated delipidation of ATG8, an essential mechanism of the autophagic cascade. Interestingly, in our glioma series, out of the 34 GBM cases which showed high immunohistochemical expression of SRSF1, high ATG7 and ATG4 levels were found in 27/34 (79%) and 23/34 (68%) cases, respectively; among the 15 cases of oligodendroglioma with high SRSF1 immunoexpression, 11/15 cases (73%) had also high levels of ATG7. ATG4 was not expressed in any oligodendroglioma case. Based on our findings, the correlation between SRSF1 and autophagy in adult gliomas remains controversial, as SRSF1 levels seemed to correlate to ATG7 and ATG4 in GBM samples and to ATG7 in oligodendrogliomas; conversely, no correlation between SRSF1 and ATG4 was found in oligodendrogliomas. Further studies with a larger cohort of cases and a wider autophagic immunohistochemical panel are required to better clarify the potential correlation between SRSF1 and autophagy in gliomas.

## 5. Conclusions

Finally, our study strongly emphasizes the role of SRSF1 as a diagnostic immunomarker of adult diffuse astrocytomas and oligodendrogliomas; in particular, the detection of increased immunohistochemical expression of this protein might be a promising and useful marker in excluding entities that frequently enter in the differential diagnosis of adult gliomas, such as ependymomas and pilocytic astrocytoma; thus, we encourage the diagnostic use of SRSF1 in some specific clinico-pathological settings in neuropathological practice.

## Figures and Tables

**Table 1:** Summary of the clinico-pathological, molecular and immunohistochemical findings.

Diagnosis/Number of Cases	Sex	Mean Age (Years)	Anatomic Site	WHO Grade	Molecular Features	SRSF1 IRS
GBM (*n* = 42)	19M; 23F	63	Temporal L (*n* = 23); parietal L (*n* = 10); frontal L (*n* = 9)	Grade IV (*n* = 42)	IDH1/2, ATRX, TP53 mutations (*n* = 0); 1p/19q co-deletion (*n* = 0); MGMT promoter non methylated (*n* = 42)	IRS ≥ 6 (*n* = 34) IRS < 6 (*n* = 8)
Oligodendroglioma (*n* = 21)	12M; 9F	59	Temporal L (*n* = 8); parietal L (*n* = 7); frontal L (*n* = 6)	Grade II (*n* = 15; grade III (*n* = 6)	IDH1 (R132H) mutations (*n* = 21); 1p/19q co-deletion (*n* = 21)	IRS ≥ 6 (*n* = 15) IRS < 6 (*n* = 6)
Ependymoma (*n* = 15)	9M; 6F	56	Supratentorial location (*n* = 11); posterior fossa (*n* = 2); spinal cord (*n* = 2)	Grade II (*n* = 14); grade I (*n* = 1)	IDH1/2, ATRX, TP53 mutations (*n* = 0); 1p/19q co-deletion (*n* = 0)	IRS ≥ 6 (*n* = 0); IRS < 6 (*n* = 2); IRS = 0 (*n* = 13)
PA (*n* = 15)	11M; 4F	23	Cerebellum (*n* = 14); spinal cord (*n* = 1)	Grade I (*n* = 15)	KIAA1549-BRAF fusion (*n* = 15)	IRS ≥ 6 (*n* = 0); IRS < 6 (*n* = 5); IRS = 0 (*n* = 10)
SEGA (*n* = 5)	3M; 2F	21	Lateral ventricles (*n* = 5)	Grade I (*n* = 5)	IDH1/2, ATRX, TP53, BRAFV600E mutations (*n* = 0); 1p/19q co-deletion (*n* = 0)	IRS ≥ 6 (*n* = 4); IRS < 6 (*n* = 1);
PXA (*n* = 4)	1M; 3F	26	Temporal L (*n* = 3); parietal L (*n* = 1)	Grade II (*n* = 3); grade III (*n* = 1)	BRAFV600E mutations (*n* = 2)	IRS ≥ 6 (*n* = 3); IRS < 6 (*n* = 1);

Abbreviations: GBM, glioblastoma; PA, pilocytic astrocytoma; SEGA, sub-ependymal giant cell astrocytoma; PXA, pleomorphic xanthoastrocytoma; M, males; F, females; L, lobe; IRS, immunoreactivity score; MGMT, O^6^-methylguanine DNA methyltransferase.

## Data Availability

No new data were created or analyzed in this study. Data sharing is not applicable to this article.
